# Supplementary figures and images for: Mannose and glycine: Metabolites with potentially causal implications in chronic kidney disease pathogenesis
Source: PLoS One. 2024 Feb 14;19(2):e0298729. doi: 10.1371/journal.pone.0298729 (PMC10866514; doi:10.1371/journal.pone.0298729)

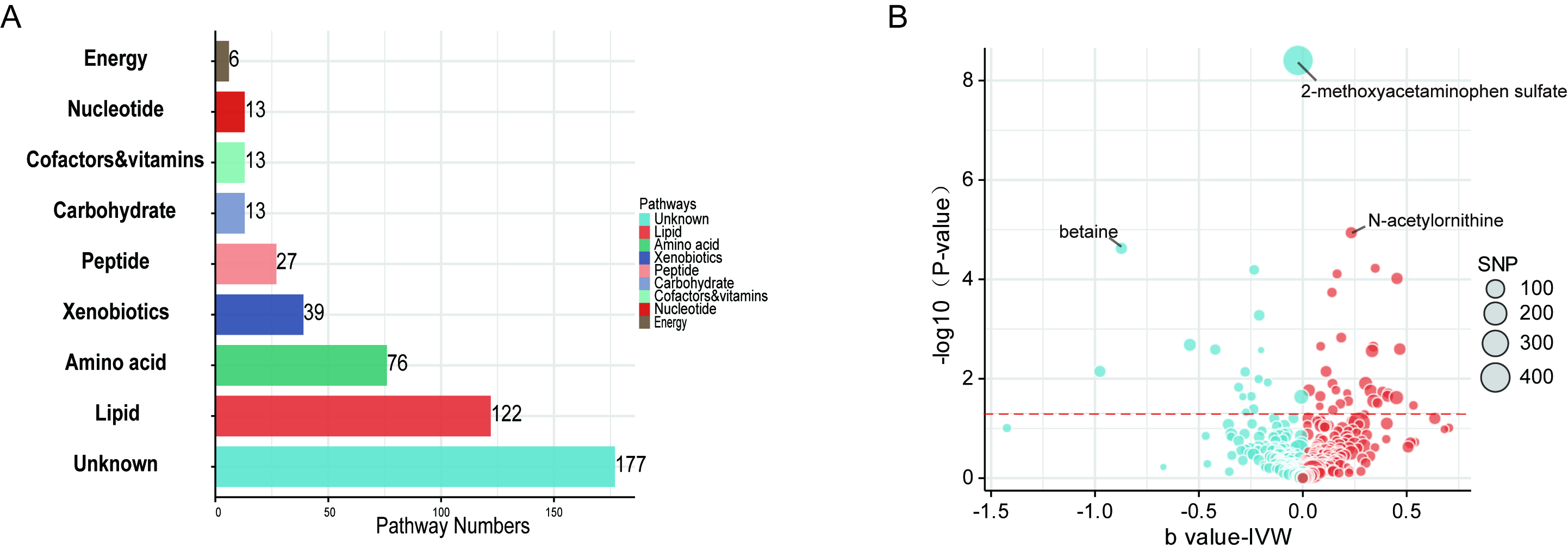

Supplement: S1 Fig — (A) Bar chart displaying the distribution of metabolites, categorized and color-coded by their respective classes. (B) A volcano plot visualizing the relationship between metabolites and CKD as assessed by the IVW algorithm. Individual dots signify specific metabolites, with the dot size reflecting the count of associated SNPs for each metabolite. Red dots indicate a positive association with CKD, while blue signifies a negative association. The red dashed line demarcates the statistical significance threshold (P = 0.05). IVW: inverse variance weighted; SNP: single nucleotide polymorphism; CKD: chronic kidney disease. (TIF) [file pone.0298729.s001.tif]

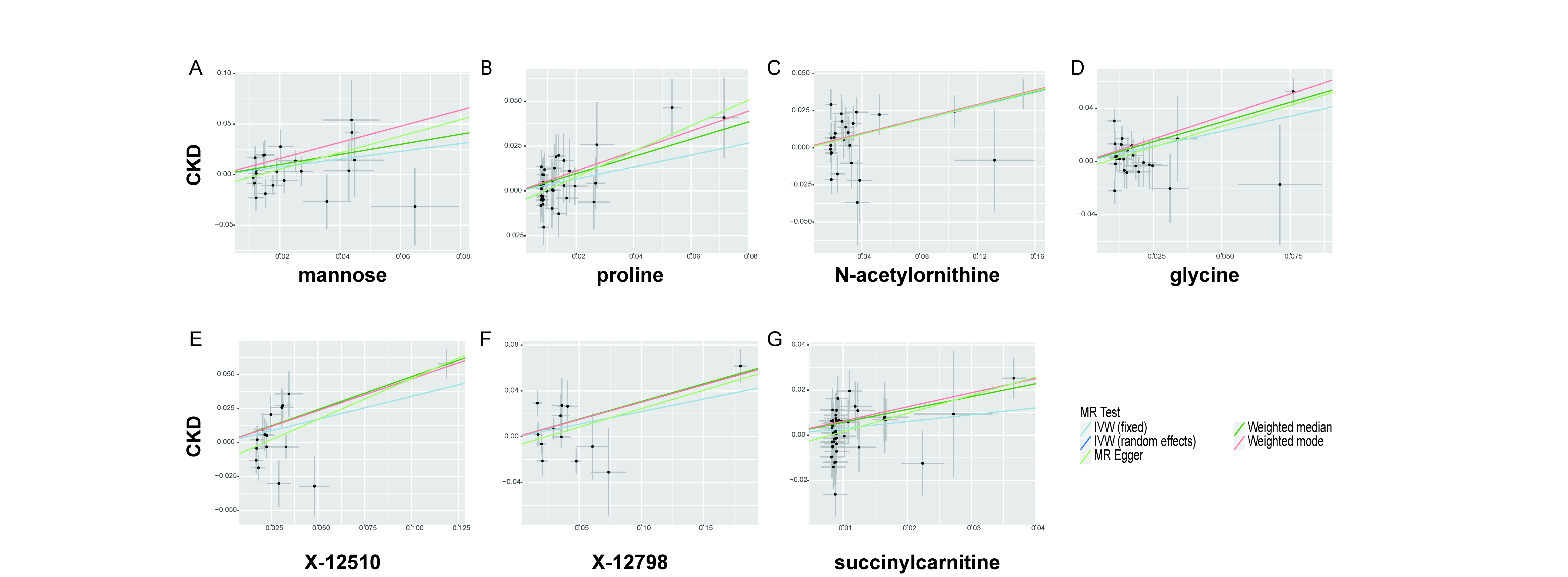

Supplement: S3 Fig — IVW: inverse variance weighted; CKD: chronic kidney disease. (TIF) [file pone.0298729.s003.tif]

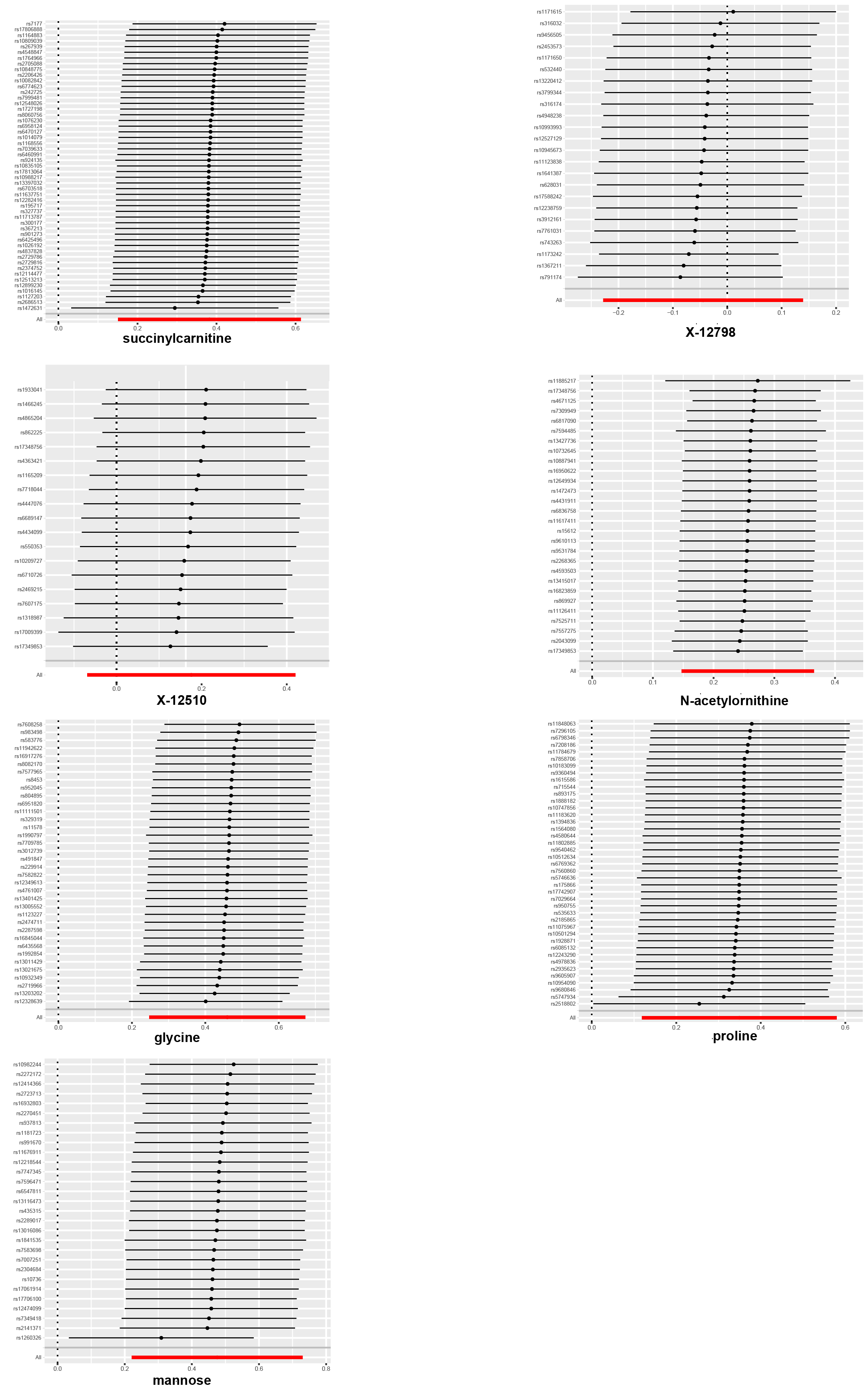

Supplement: S4 Fig — (TIF) [file pone.0298729.s004.tif]

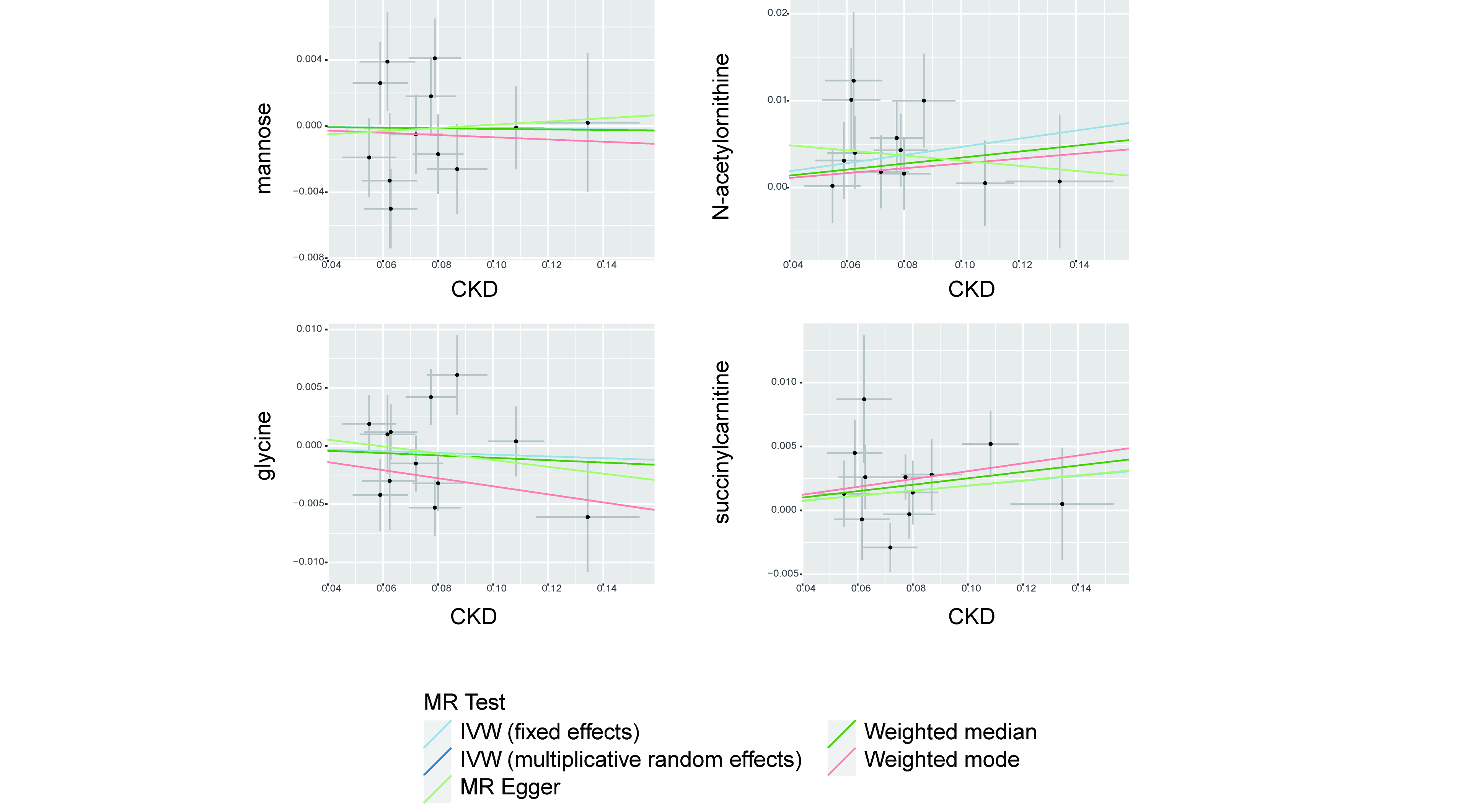

Supplement: S5 Fig — IVW: inverse variance weighted; CKD: chronic kidney disease. (TIF) [file pone.0298729.s005.tif]

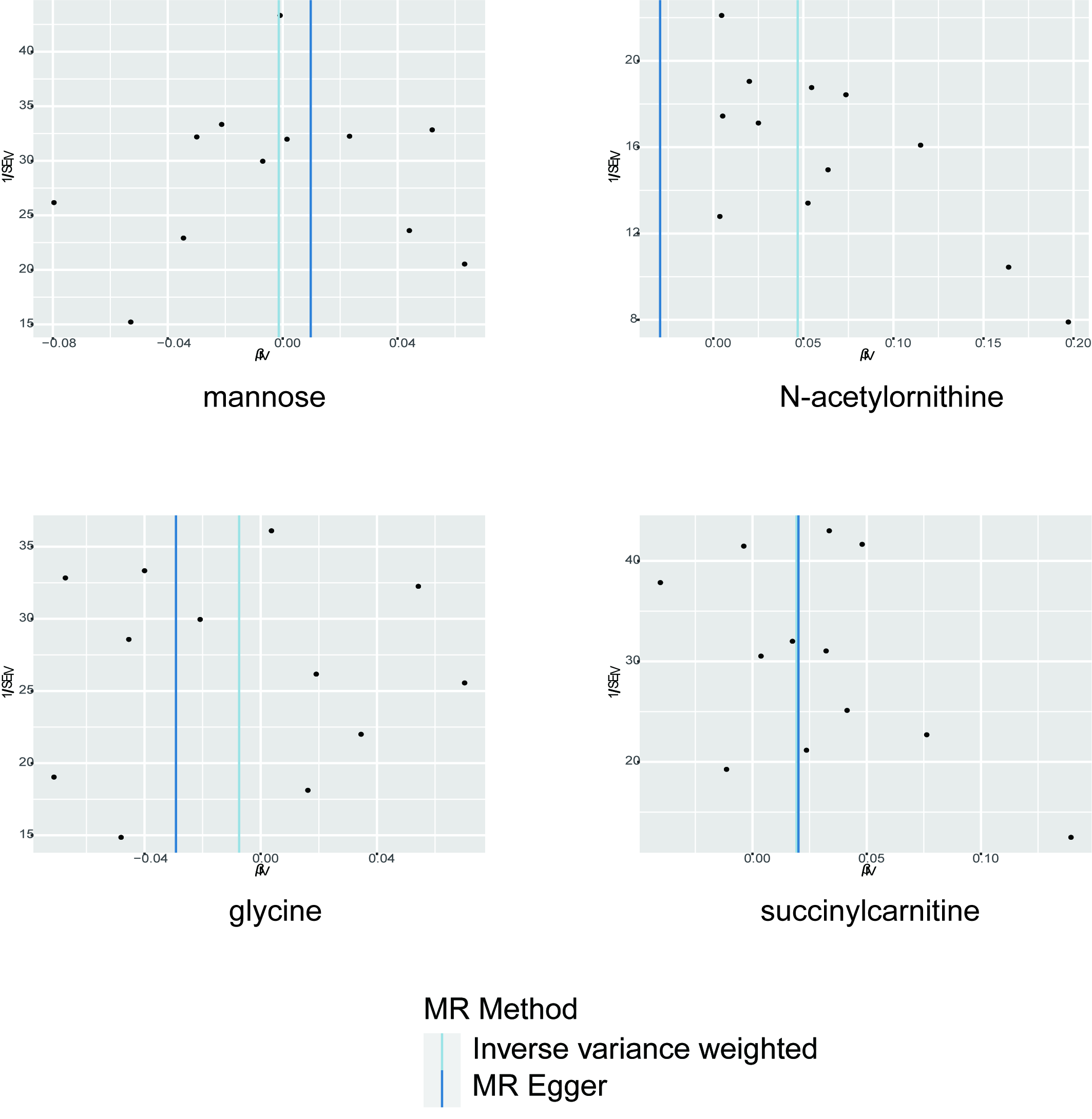

Supplement: S6 Fig — CKD: chronic kidney disease. (TIF) [file pone.0298729.s006.tif]

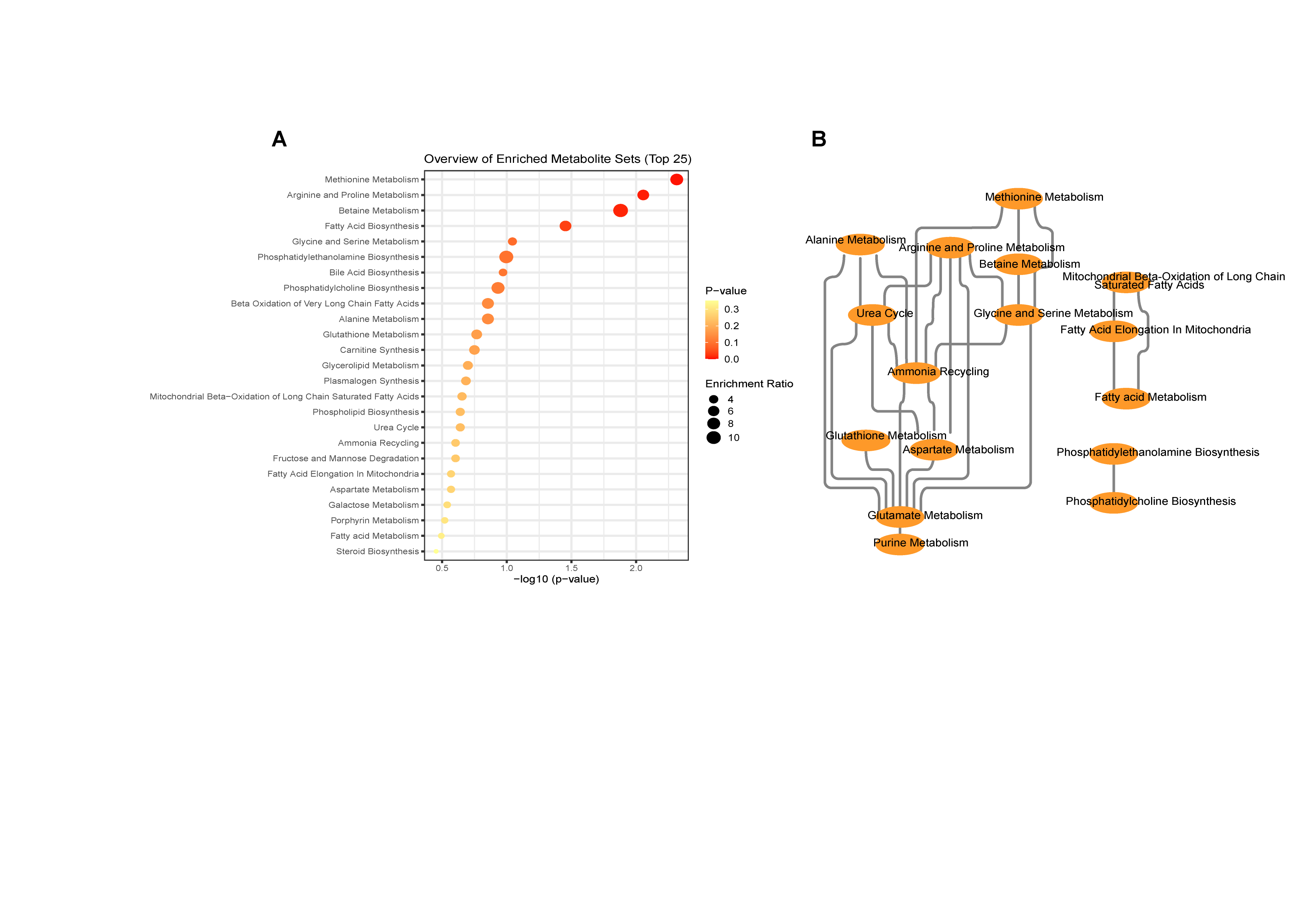

Supplement: S7 Fig — (A) Dot plots show the top 25 metabolic pathways in which significant metabolites participate, selected by the IVW algorithm. (B) Network diagram, individual nodes denote distinct metabolite sets. An edge or a connection between two metabolite sets indicates that they have an overlap, wherein more than 25% of their metabolites are shared. (TIF) [file pone.0298729.s007.tif]
