# Supplementary material for: Mannose and glycine: Metabolites with potentially causal implications in chronic kidney disease pathogenesis
Source: PLoS One. 2024 Feb 14;19(2):e0298729. doi: 10.1371/journal.pone.0298729 (PMC10866514; doi:10.1371/journal.pone.0298729)
Supplement: Attachment — Submitted filename: Response to Reviewers_2.docx [file pone.0298729.s016.docx]

**Response to Journal**

Thank you for your time and attention to our manuscript. We understand that PLOS offers authors the option to publish the peer review history of their articles. After careful consideration, we would like to express our consent to publish the full peer review history associated with our manuscript, should it be accepted for publication.

We appreciate the efforts and insights provided by the peer reviewers throughout the review process. The constructive feedback has significantly contributed to enhancing the quality of our research. We believe that making this peer review history publicly available will not only promote transparency but also serve as an educational resource for the scientific community.

**Response to Reviewers**

We would like to extend our heartfelt gratitude for your insightful and constructive feedback on our manuscript. Your detailed reviews and valuable suggestions have significantly contributed to enhancing the quality and rigor of our work. Thank you for your time and efforts in guiding us through this revision process.

**For Reviewer #1** Giacomo Garibotto

*“Thank you for revising your MS. in accordance with changes made in the MS I believe that a few issues still need to be addressed:
Title: “Mannose and Glycine: metabolites with causal implications in chronic kidney disease pathogenesis” I would suggest to modify as: “Mannose and Glycine: metabolites with potentially causal implications in chronic kidney disease pathogenesis”.Similarly, sentences in the figures also need to be changed. Please also check spelling in Figure 1 (casual vs. causal).”*

Thank you very much for your thorough review and valuable suggestions regarding our manuscript. We have carefully considered your feedback and have accordingly revised the title of our paper to “Mannose and Glycine: metabolites with potentially causal implications in chronic kidney disease pathogenesis.” This modification better reflects the probabilistic nature of our findings.

In addition, we have revised the wording in Figure 1 to align with the suggested changes, ensuring that our descriptions accurately represent the nature of our findings. We are also grateful for your attention to detail in identifying the spelling error (“casual” instead of “causal”). This has been corrected in the revised version of the figure.

Your guidance has been instrumental in enhancing the clarity and accuracy of our work, and we deeply appreciate your contributions to improving the quality of our manuscript.

**For Reviewer #2** Matteo Breno

*“While the authors provided more details on the methods, two of the main concerns have not been addressed.The first is the effect of single influential variants (see comment below).The second is the issue of multiple testing. The authors tested 486 metabolites and they did not perform a correction for multiple tests (see also comment below) without offering a reason.”*

We sincerely appreciate your continued guidance and constructive feedback on our manuscript. Your insights are invaluable in refining our research methodology and presentation. We hope the additional details and explanations below will address your concerns and further reinforce the validity and reliability of our findings. Thank you again for your thorough review and invaluable feedback.

*“- Methods, line 112. I think you should you clearly state that you used a threshold of 1e-5 because the number of variants associated with metabolites at genome-wide level was low. Especially because you used a more stringent threshold for CKD.”*

Thank you for your insightful comment regarding the threshold used for selecting instrumental variables (IVs) in our study. We appreciate the opportunity to clarify this aspect of our methodology in the manuscript.

As per your suggestion, we have made a more explicit statement in the Methods section (now reflected in Line 109) to elucidate our rationale for using a p-value threshold of 1×10^−5 for selecting IVs associated with metabolites. We acknowledge that this threshold is less stringent compared to the threshold used for CKD-related genetic variants. This approach was adopted due to the relatively low number of variants associated with metabolites at the genome-wide significance level. In contrast, for CKD, where a larger number of genome-wide significant variants are available, a more stringent threshold of 5×10^−8 was employed to ensure the robustness of our results.

We trust that this clarification enhances the understanding of our methodological choices and addresses your concern. We are grateful for your valuable feedback and are committed to ensuring the highest level of clarity and precision in our manuscript.

*“- Results, line 179. The sentence "suggesting a likely positive correlation with CKD" is redundant since you selected only the positive relationships.”*

Thank you for your attentive review of the Results section of our manuscript. We agree with your observation regarding the redundancy in the sentence "suggesting a likely positive correlation with CKD," particularly in the context where we selected only the positive relationships.

In accordance with your suggestion, we have revised the manuscript to remove this redundancy. This edit can be found in Line 186 of the Results section. We appreciate your guidance in enhancing the clarity and conciseness of our manuscript. We are committed to upholding the highest standards of academic rigor and precision in our reporting.

*“- Results, line 180. The sensitivity analyses done in MR studies do not address the issue of multiple testing.”*

Thank you for your detailed review and for highlighting the issue of multiple testing in our MR analysis. Your comment points to a crucial aspect of our study, namely, the appropriate adjustment for multiple comparisons when conducting MR analyses.

In response to your suggestion, we have reanalyzed our data using the IVW method with the application of the False Discovery Rate (FDR) to correct for multiple testing. This correction method is aimed at reducing false-positive findings that may arise from conducting multiple comparisons. After the FDR adjustment, our results still support, to some extent, our previous findings indicating potential causal relationships between certain metabolites and CKD. However, we also recognize that FDR adjustment may lead to some associations previously deemed significant no longer being significant under this stricter criterion. The full FDR results have been uploaded to the file *“****mendelian test_FDR.xls****”*

Consequently, in our discussion section, we have specifically emphasized the importance of appropriate adjustment for multiple testing in MR analyses and acknowledged this as a limitation of our study (line 349). We have added more detailed information in the limitations section of our discussion to address this point, highlighting the potential improvements for future studies in this area.

We believe these adjustments and additions will enhance the overall quality and robustness of our research and are grateful for your valuable feedback.

*“- Results, line 198. I do not think you addressed the issue of single influential markers. For example, in the first forest plot of figure S4, when the variant rs1260326 is removed the confidence intervals largely overlap 0. When this variant is retained, and the others are removed one by one, the effect become significant. This clearly indicate that the results for mannose are driven by the variant rs1260326. This hold for all metabolites, but N-acetylornithine.”*

Thank you for your astute observation regarding the issue of single influential markers in our Mendelian Randomization (MR) analysis, specifically concerning the variant rs1260326 and its impact on the results for mannose. We agree with your assessment and appreciate your attention to this detail.

In response to your comment, we conducted a reanalysis during our first revision, where we meticulously excluded potential influential SNPs and subsequently redrawn the forest plots. These revised analyses are presented in the file titled “***S.F.4 leave-one-out_new.***” It appears that there may have been an oversight in file categorization, and we apologize if this has caused any confusion. The redrawn forest plot, which addresses your concern, was uploaded under the “other file” section, which might be why it was not immediately noticeable in your review.

We believe that this reanalysis strengthens the validity of our results and provides a more accurate representation of the data. We hope that this additional information adequately addresses your concerns. Thank you once again for your valuable feedback and guidance.

*“- Results, Metabolic pathway analysis. I did not understand which metabolites were actually used for the pathway analysis and by which criteria you did select them.”*

Thank you for your query regarding the metabolic pathway analysis in our study. We understand the importance of clarity in describing the selection criteria for metabolites used in such analyses.

In our study, we selected 32 metabolites for the pathway analysis based on the criterion of a p-value < 0.05 using the Inverse Variance Weighted (IVW) method. The details of these selected metabolites can be found in the supplementary file “***Table S6 Pathways involved metabolites***” which lists all the metabolites included in the pathway analysis.

Following the selection, we utilized the online platform Metaconfict 5.0 for analyzing these 32 metabolites. Of these, 14 metabolites were effectively identified and recognized by the platform. It is these 14 identified metabolites that were then used for downstream pathway analysis.

We hope this explanation clarifies the selection process for the metabolites used in our pathway analysis and addresses your query. Thank you again for your insightful feedback.

*“*- Line 191. The title should be "Sensitivity analysis"

Thank you for your careful reading and constructive feedback on our manuscript. We have taken your suggestion into account and amended the title of the section from "Sensitive Analysis" to "Sensitivity Analysis." This change has been made in Line 198 of the manuscript.
