# Supplementary material for: Mannose and glycine: Metabolites with potentially causal implications in chronic kidney disease pathogenesis
Source: PLoS One. 2024 Feb 14;19(2):e0298729. doi: 10.1371/journal.pone.0298729 (PMC10866514; doi:10.1371/journal.pone.0298729)
Supplement: Attachment — Submitted filename: Response to Reviewers_3.docx [file pone.0298729.s017.docx]

**Response to Journal**

We would like to extend our gratitude for the thorough review and management of our manuscript submission process. We acknowledge the opportunity provided by PLOS to share the peer review history of articles, and after careful consideration, we agree to this initiative for our manuscript, should it be favorably accepted for publication.

The insightful and detailed feedback received from the peer reviewers has been instrumental in refining our work. Their expertise and constructive suggestions have undoubtedly elevated the quality of our research. By making the peer review history public, we aim to contribute to the spirit of transparency and open scientific discourse, which we believe are essential for the advancement of knowledge.

Thank you once again for facilitating this process, and we look forward to the potential impact that publishing the peer review history could have on the scientific and academic community.

**Response to Reviewers**

We are deeply thankful for your valuable and insightful feedback regarding our manuscript. Your comprehensive reviews and thoughtful suggestions have played a crucial role in substantially improving both the quality and the depth of our research. We sincerely appreciate the time and effort you have dedicated to meticulously guiding us through the process of revising our manuscript.

**For Reviewer #1** Giacomo Garibotto

*“The authors have not indicated how to retrieve data and calculations (see data availability statement)”*

Thank you for your valuable feedback on our manuscript. We appreciate the opportunity to clarify the sources and accessibility of the data used in our Mendelian Randomization study.

Our MR analysis is fundamentally based on publicly accessible databases. Specifically, the metabolite data were sourced from the Metabolomics GWAS Server, accessible at https://metabolomips.org/gwas/. The chronic kidney disease data were obtained from the CKDGen Consortium, available at http://ckdgen.imbi.uni-freiburg.de/. Both these sources are open to the public and have played a crucial role in our study. We have cited the original publications associated with these data in the reference section of our manuscript. This ensures that readers have complete information on the origin and context of the data used.

In the Methods section of our manuscript, we have elaborately described the experimental design methodology of our MR study. We have also detailed the specific versions of R and its packages used for our analysis. This was done with the intention of aiding other researchers in replicating or building upon our MR study results.

Our metabolic pathway analysis is conducted using the publicly available online data analysis platform, which can be accessed at https://www.metaboanalyst.ca/. We believe that mentioning these resources explicitly in our manuscript adds value by highlighting the integral role these databases have played in our study.

We understand the importance of transparency and reproducibility in scientific research and have made sure that our manuscript reflects this by providing clear information on data sources and methodologies. We will ensure that these details are prominently included in the revised manuscript for the benefit of the reviewers and the broader scientific community.

Thank you once again for your guidance in improving our manuscript.

**For Reviewer #2** Matteo Breno

*“The file mendelian test_FDR.xls is not available to me, however I suggest to add it as supplementary table and to explicitly mention it in the results together with a sentence addressing the multiple tests issue (similar to what you wrote in the Response to Reviewers).”*

Thank you for your continued attention to our manuscript and valuable feedback. In response to your suggestion, we have re-uploaded the file "mendelian test_FDR.xls" to the supplementary files section as "S7 Table." Additionally, we have included a specific mention of this table in the Results section of our manuscript, along with a sentence addressing the issue of multiple testing.

In the manuscript, at Line 189, we have now added the following text to provide further clarity:

"After applying the false discovery rate (FDR) correction to adjust for multiple comparisons, our analysis continues to moderately support our initial findings, suggesting possible causal links between specific metabolites and CKD. The FDR-adjusted results are presented in Supplementary files."

We believe this addition will enhance the transparency and rigor of our study, and we are grateful for your guidance in improving the quality of our manuscript.
